# Supplementary material for: Impairment of visually guided associative learning in children with Tourette syndrome
Source: PLoS One. 2020 Jun 16;15(6):e0234724. doi: 10.1371/journal.pone.0234724 (PMC7297359; doi:10.1371/journal.pone.0234724)
Supplement: Attachment — Submitted filename: PLoS One review due 13 Feb.pdf [file pone.0234724.s002.pdf]

The manuscript reports the results of a study comparing the associative learning performance of children with Tourette syndrome (TS) in the absence of any comorbidity (N=21) with children with TS and ADHD (N=15), on associative learning in a variant of the Rutgers Acquired Equivalence Test (adapted for use in Hungarian children). N=36 matched controls were also tested. Children with TS comorbid with ASD or OCD (N=11) are mentioned but I can't see that these data have been included. These could well be publishable findings but I'm struggling to follow the conclusions in the present version of the manuscript.

Points to be addressed

As is to be expected for this kind of work, the sample sizes are reasonable, but likely insufficient to take any differences attributable to medication into account. N=3 TS and N=6 TS+ADHD children were medicated and medication has previously been found to affect associative learning in both TS and ADHD. I'm not sure that the medication details have been provided and I can't see that potentially confounded effects of medication have been discussed at all within the present manuscript.

If the lack of difference in performance between TS and TS+ADHD groups suggests that the effects shown in Figure 2 (differences in NAT and ALER) are TS-mediated, should the same profile not also be shown in the 3rd TS group (comorbid with ASD or OCD)?

The third TS group don't seem to be shown in the figures, nor are the data considered with and without the exclusion of participants on medication?

Ln 123-128 - I didn't understand the rationale to categorise IQ scores: like age, IQ is a continuous variable and to categorise the participants' scores into IQ ranges may result in a loss of statistical power. Please explain. It's also not clear how we are to understand the categories which run from 'extremely high' through to 'very low' with no further definition of these categories.

Ln 361-365 – I can't see how this conclusion about the compensatory effect of ADHD follows. I thought the TS and TS+ADHD groups were not significantly different from each other? Now we're getting a different comparison (each of the sub-groups with the controls) and the TS-ADHD group also happens to be smaller (N=15). Plus medication does not seem to have been taken into account.

The writing is mostly understandable but the manuscript would benefit from further editing by a fluent English speaker, this is not really the job of the reviewer. For example, the Abstract which is most visible (and all some will read) could be better written (some specific suggestions below).

Ln 21-22 - The first sentence is a little awkward.
Ln22-23 – 'the majority of the cognitive functions' - 'the majority of cognitive functions'
Ln 23 - 'only little evidence' – suggest rephrase
Ln 26 – 'The acquired equivalence learning…' - 'Acquired equivalence learning…'
Ln 32 – 'the entire patient group' – please be more specific, presumably TS+ADHD
Ln 33 – 'associations with lower effectiveness' – please rephrase
Ln 36-37 – 'parts of the test phase' – plural so 'depend…' (and sentence seems to be missing a comma after hippocampus)

Minor comments and typos

Ln 140 - iOS – should be defined?

Ln 184 – misplaced (and uninformative) figure caption text.

Ln 211-212 – Data availability statement and link will need updating. I'm not sure I see the point of providing the data for peer review only at the first revision if the data have not been provided at this point (for peer review prior to the point of being on a 'revise and resubmit' ticket)? Elsewhere (in the additional information boxes) it says all relevant data are within the manuscript and its Supporting Information files but I can't see that the data has in fact been provided at this point. The Supporting Information files seem to be figures.

Ln 238-248 – I think this para is the figure legend.

Ln 272-277 – I think this para is the figure legend.

Ln 297-302 – I think this para is the figure legend (ditto for other figures, why is the legend in the text?)

Ln 579 – please make the figure caption self-explanatory rather than referring the reader back to the text.

Ln 583 - are the data for TS and TS+ADHD participants in fact shown separately in Figure 2? (As implied by the figure header.) If we're looking at all the TS data relative to matched controls why not also include TS+OCD/ASD?

Ln 608 - 'syndrom' (typo)

Ln 612 - please make the figure caption self-explanatory rather than referring the reader back to an earlier figure legend.
