# Supplementary material for: Impairment of visually guided associative learning in children with Tourette syndrome
Source: PLoS One. 2020 Jun 16;15(6):e0234724. doi: 10.1371/journal.pone.0234724 (PMC7297359; doi:10.1371/journal.pone.0234724)
Supplement: Attachment — Submitted filename: Response to Reviewers2.docx [file pone.0234724.s004.docx]

First of all, we would like to express our gratitude for the scholarly and highly helpful criticism of the Academic Editor and the Referees, which helped us to improve the quality of our study. We accepted all of the suggestions made changes accordingly. The suggestions are answered below itemized; textual changes are marked in the final re-revised manuscript.

Additional Editor Comments (if provided):

Thank you for the corrections performed to the original version of the submission. Most of the issues raised by the Reviewers were addressed; there is still a question of the statistical analyses (e.g. correction for multiple comparisons, when comparing different subgroups (with/nomedication, without/with different comorbidities) with their controls), making it hard to solidly base the conclusions on the obtained results.

**Answer: In the case of the entire (medicated and unmedicated) TS patient group and the entire unmedicated TS group we have applied the same data in the statistical analysis. Because of this double application of the data we have performed the Bonferroni correction of the statistical results. The new significance level (p=0.025) was applied but the results remain unchanged, the differences remain after the correction similarly significant between the TS and control groups in both cases of NAT and ALER. Because of the no significant differences concerning RER and GER the further correction is not essential.**

**We have additionally performed a summarized comparison of three TS patient and the three control subgroups (Kruskal-Wallis ANOVA), which revealed statistically significant differences among the six subgroups in NAT and ALER but not in RER and GER. In order to see the origin of these significant differences in NAT and ALER we have compared in the next step the performances of the three TS subgroups and the performances of the three control subgroups, respectively. There were no significant differences in the performances among the TS subgroups and there were no significant differences among the control subgroups. Thus the difference could be only because of the differences between the particular TS subgroups and their pairwise matched controls (see the results in the last three chapters of the Results).**

Reviewer #1: Thank you for the substantial revisions in response to Reviewer comments. Several additional comments are below.

**We can’t understand the concern of the reviewer about the data availability, because we have uploaded in the previous (revised) draft of the manuscript the entire xls table, which contains the psychophysical performances of all patients and controls in the supplementary materials of the manuscript.**

Major Comments:

1. Does age differ significantly among subgroups? For example, is the nearly one-year age difference between the TS+ADHD and TS+OCD/ASD groups (patients or controls) significant?

**Response: The age was not different among the TS patient subgroups. We have added this statement to the manuscript. The age was not different among the control subgroups, too.**

2. Does tic severity differ significantly among patient subgroups? Increased tic severity can accompany comorbid conditions.

**Response: The tic severity was not different among the TS patient subgroups. We have added this statement to the manuscript.**

3. Does IQ differ significantly among patient subgroups? Of note, tic severity and IQ do not need to be included in Table 1, but a statement can be made that they do not differ among subgroups, if that is the case.

**Response: The IQ was not different among the TS patient subgroups. We have added this statement to the manuscript. The IQ was not different among the control subgroups, too.**

4. Does performance differ significantly among control subgroups? A pitfall of enrolling one control per patient (rather than the same 10-20 controls used for all subgroups) is that the control subgroups may differ among themselves. Whereas age and gender appear to match, task performance varies. Specifically, the TS+ADHD controls had a median NAT of 75, whereas the other control subgroups had median NATs of 60.5 and 60, respectively. The TS+ADHD controls had a median ALER of 0.105, whereas the other control subgroups both had median ALER scores of 0.083. This potentially significant difference among control subgroups is critical, for it could be the reason the TS+ADHD patient group does not differ from controls. This would change the interpretation of the data from ADHD not altering cognitive functions, to indeed altering visual acquisition.

**Response: Thank You for this important question. None of the performances (NAT, ALEG, RER, GER) were different among the control subgroups. We added these results to the manuscript.**

Minor Comments:

1. On line 193, what is meant by “involuntary…attention”? Involuntary attention is to a unattended, unexpected stimulus that reflexively draws the subject’s gaze. “Undivided attention” sounds more like voluntary (albeit undivided) attention.

2. The task description in the Methods is far more clear. Thank you.

3. On line 233, reverse the order of words to read “with and without.”

4. On line 306, please delete the word “After.”

5. In Tables 2 and 3, add an “n” in “range” for the ALER of patients with TS.

6. On line 369, what alterations are referenced? Perhaps “significant findings”?

7. In the Results, please place the data of patients with TS+ADHD before that of TS+OCD to mirror all preceding text and tables.

**Answer: We accepted all of these minor suggestions and corrected the manuscript accordingly.**
